# Inhibition of mTOR signaling and clinical activity of metformin in oral premalignant lesions

**DOI:** 10.1172/jci.insight.147096

**Published:** 2021-09-08

**Authors:** J. Silvio Gutkind, Alfredo A. Molinolo, Xingyu Wu, Zhiyong Wang, Daniela Nachmanson, Olivier Harismendy, Ludmil B. Alexandrov, Beverly R. Wuertz, Frank G. Ondrey, Denise Laronde, Leigha D. Rock, Miriam Rosin, Charles Coffey, Valerie D. Butler, Lisa Bengtson, Chiu-Hsieh Hsu, Julie E. Bauman, Stephen M. Hewitt, Ezra E.W. Cohen, H-H. Sherry Chow, Scott M. Lippman, Eva Szabo

**Affiliations:** 1 Moores Cancer Center, University of California, San Diego (UCSD), La Jolla, California, USA.; 2 Department of Pharmacology, UCSD School of Medicine, La Jolla, California, USA.; 3 Bioinformatics and Systems Biology Graduate Program, UCSD, La Jolla, California, USA.; 4 Division of Biomedical Informatics, Department of Medicine, UCSD School of Medicine, La Jolla, California, USA.; 5 Department of Otolaryngology, Head and Neck Surgery, University of Minnesota (UMN), Minneapolis, Minnesota, USA.; 6 Department of Oral Biological and Medical Sciences, Faculty of Dentistry, University of British Columbia, Vancouver, British Columbia, Canada.; 7 British Columbia Cancer Agency (BCCA), British Columbia Agency Research Center, Vancouver, British Columbia, Canada.; 8 University of Arizona Cancer Center, Tucson, Arizona, USA.; 9 Division of Cancer Prevention and; 10 Center for Cancer Research, NCI, Bethesda, Maryland, USA.

**Keywords:** Clinical Trials, Head and neck cancer, Signal transduction

## Abstract

**BACKGROUND:**

The aberrant activation of the PI3K/mTOR signaling circuitry is one of the most frequently dysregulated signaling events in head and neck squamous cell carcinoma (HNSCC). Here, we conducted a single-arm, open-label phase IIa clinical trial in individuals with oral premalignant lesions (OPLs) to explore the potential of metformin to target PI3K/mTOR signaling for HNSCC prevention.

**METHODS:**

Individuals with OPLs, but who were otherwise healthy and without diabetes, underwent pretreatment and posttreatment clinical exam and biopsy. Participants received metformin for 12 weeks (week 1, 500 mg; week 2, 1000 mg; weeks 3–12, 2000 mg daily). Pretreatment and posttreatment biopsies, saliva, and blood were obtained for biomarker analysis, including IHC assessment of mTOR signaling and exome sequencing.

**RESULTS:**

Twenty-three participants were evaluable for response. The clinical response rate (defined as a ≥50% reduction in lesion size) was 17%. Although lower than the proposed threshold for favorable clinical response, the histological response rate (improvement in histological grade) was 60%, including 17% complete responses and 43% partial responses. Logistic regression analysis revealed that when compared with never smokers, current and former smokers had statistically significantly increased histological responses (*P* = 0.016). Remarkably, a significant correlation existed between decreased mTOR activity (pS6 IHC staining) in the basal epithelial layers of OPLs and the histological (*P* = 0.04) and clinical (*P* = 0.01) responses.

**CONCLUSION:**

To our knowledge this is the first phase II trial of metformin in individuals with OPLs, providing evidence that metformin administration results in encouraging histological responses and mTOR pathway modulation, thus supporting its further investigation as a chemopreventive agent.

**TRIAL REGISTRATION:**

NCT02581137

**FUNDING:**

NIH contract HHSN261201200031I, grants R01DE026644 and R01DE026870

## Graphical abstract

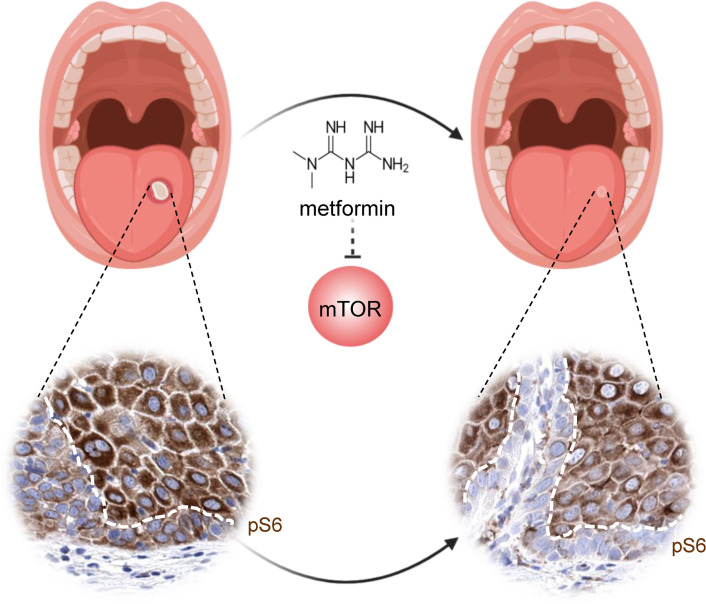

## Introduction

Every year more than 600,000 cases of head and neck squamous cell carcinoma (HNSCC), which arise in the oral cavity, oropharynx, larynx, and hypopharynx, are diagnosed worldwide, ranking sixth overall in incidence (1). In the United States, more than 65,000 new cases of HNSCC were predicted to occur in 2020, resulting in 14,500 deaths (1). Despite encouraging novel therapies, only a limited improvement in the survival rates for patients with HNSCC has occurred in the last 4 decades, particularly in tongue and other oral cavity cancers that are often associated with tobacco use and alcohol consumption as the main risk factors (2). Poor treatment outcomes are generally the result of delayed diagnosis and “field cancerization,” a unique term describing the occurrence of multifocal potentially malignant lesions or second primary HNSCC (3). Clearly, prevention and early diagnosis are keys to significantly improving the prognosis of patients with HNSCC. Ten randomized clinical trials have been reported for oral cancer chemoprevention, none of which had a positive, long-term effect on cancer development. Initial studies using high doses of 13-cis-retinoic acid reduced oral premalignant lesions (OPLs) and prevented second primary tumors (4). However, chronic administration of 13-cis-retinoic was not tolerable, and although lower doses were tolerable, they were ineffective (5). Similarly, the recently reported Erlotinib Prevention of Oral Cancer trial, which was the first trial involving participant selection based on risk (6, 7), did not provide an effective targeted preventive strategy for HNSCC, specifically in individuals with OPLs, who are at a higher risk of developing HNSCC (8).

The recent elucidation of the HNSCC genomic landscape revealed that multiple genetic alterations underlie the development of this aggressive malignancy, including mutations and genetic alterations in the *TP53*, *FAT1*, *NOTCH1*, *CASP8*, *CDKN2A* (*p16*^INK4A^), and *PIK3CA* genes (9–11). In particular, *PIK3CA*, which encodes the PI3Kα catalytic subunit, is the most commonly mutated oncogene in HNSCC (~20%). This underlies our initial observations that the aberrant activation of the PI3K/mTOR signaling pathway is a widespread event in HNSCC (>80% of all HPV^–^ and HPV^+^ cases; refs. 12, 13). We also observed that mTOR inhibitors (mTORis) display potent antitumor activity in a large variety of genetically defined and chemically induced experimental HNSCC models (13–19) as well as in our recently reported phase II clinical trial in patients with HNSCC (20). This supports that PI3K/mTOR signaling may represent a druggable candidate in HNSCC. However, the potential immunosuppressive activity of direct mTORis may raise safety concerns regarding their long-term use as chemopreventive agents (21). This prompted us to focus on the potential use of metformin, which targets mTOR indirectly, for HNSCC prevention.

Metformin is an oral biguanide that is currently the drug of choice for the treatment of type 2 diabetes and is being prescribed to at least 120 million people worldwide (22). Hence, metformin’s safety profile for long-term use and the management of its potential side effects are well-documented. Metformin treatment reduces tumor cell growth in part by reducing the activity of mTOR as part of its complex, mTORC1 (23–26). In prior studies, we have shown that metformin causes a significant reduction in the conversion of OPLs into HNSCC in mice (27) and that metformin decreases mTOR activity and HNSCC progression by acting on cancer-initiating cells directly (28, 29). Specifically, the knockdown of the metformin transporter OCT3, which is highly expressed in normal and neoplastic oral epithelium, or rescuing oral cancer cells from the effects of metformin on mitochondrial complex I, nearly abolishes the antitumor activity of metformin in mice in vivo (28, 29). Aligned with these experimental studies, 2 large retrospective population case-control cohort studies together involving more than 300,000 diabetic patients demonstrated a decrease in the risk of HNSCC in individuals on metformin (30, 31). Metformin use also results in better overall survival in diabetic patients diagnosed with laryngeal HNSCC (32). Based on these experimental findings and the emerging epidemiological evidence, we developed a clinical trial (M4OC-Prevent, NCT02581137) of individuals with OPLs to explore the potential use of metformin for HNSCC prevention.

## Results

**Figure 1:**
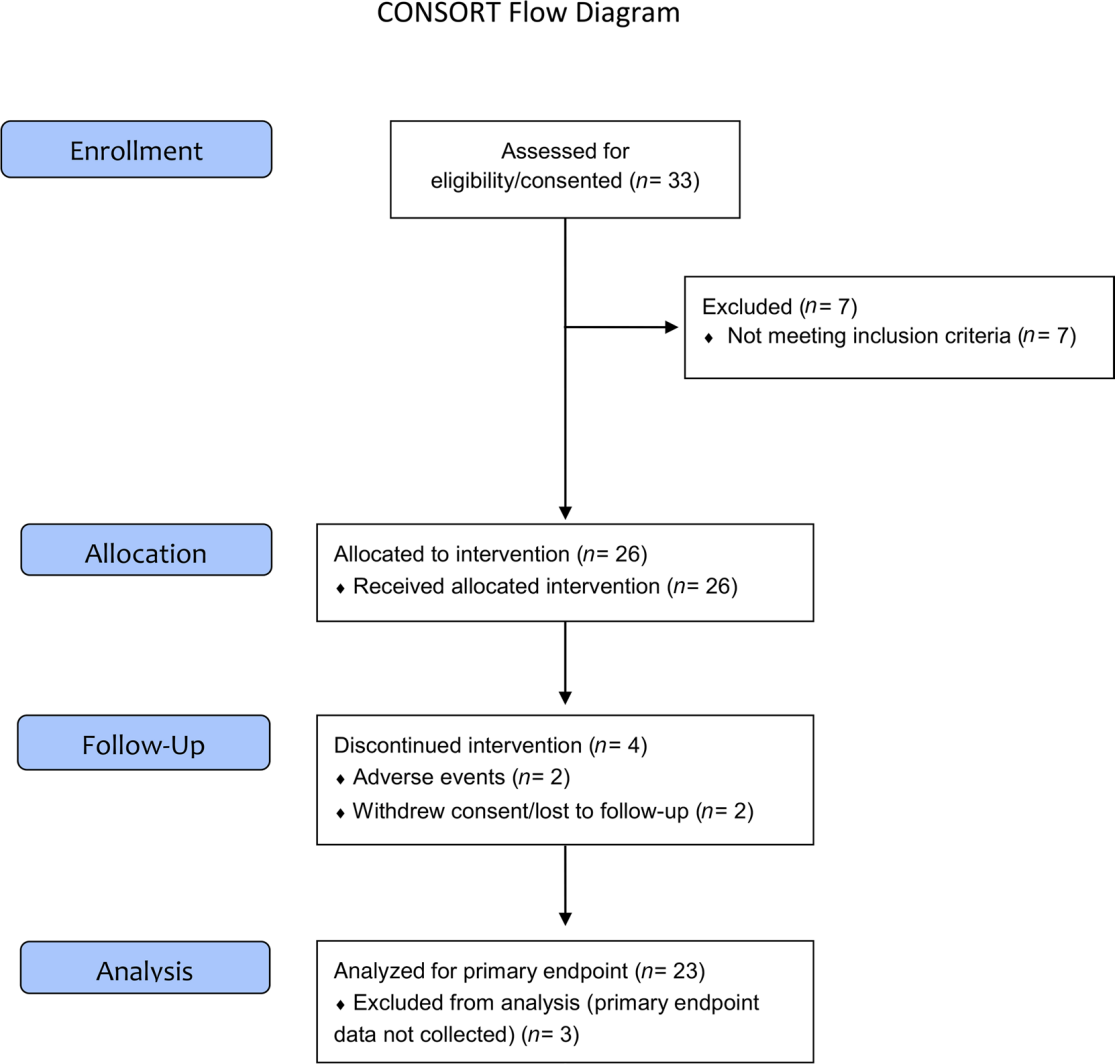
CONSORT diagram. The study (M4OC Prevent) was a phase IIa single-arm, open-label trial of individuals with oral leukoplakia or erythroplakia to explore the potential of metformin for oral cancer prevention (NCT02581137). The primary endpoint was to determine whether 12–14 weeks of metformin intervention is associated with the clinical response of OPLs. The secondary endpoints included histologic response to metformin in the target lesion, pretreatment and posttreatment tissue-based biomarkers of molecular targets and dysregulated molecular mechanisms, modulation of circulating metabolic biomarkers, and serum and saliva metformin concentrations. A brief summary of the trial workflow, schedule of baseline evaluation, intervention, and postintervention evaluation, and agent used (metformin) is depicted.

The study opened to accrual in June 2016 and closed to accrual in July 2017 (Figure 1). Thirty-three potentially eligible participants were consented: 4 from UCSD, 16 from BCCA, and 13 from UMN. Of these, 26 met all eligibility criteria and initiated agent intervention. Twenty-two participants completed the 12–14 weeks of agent intervention, and 4 participants did not complete the 12–14 weeks of agent intervention (2 due to adverse events [AEs] and 2 withdrew consent). One participant terminated intervention early but provided postintervention research specimens for outcome evaluation, which was included in the analysis. Thus, 23 participants were considered evaluable.

The demographics of participants who initiated the agent (*n* = 26) are summarized in Table 1. The average age was 58 ± 11 years. Fourteen participants were women. The average BMI from these participants was 30.1 ± 6.8 kg/m^2^. The majority were White and non-Hispanic. Former and current smokers accounted for 42% and 12%, respectively. Baseline disease characteristics of participants who provided postintervention biopsy specimens for research endpoints (*n* = 23) are also summarized in Table 1. The majority of the participants had mild/moderate dysplasia (87% combined). No erythroplakia lesions were included in the study. The lesions were mostly found on the tongue (70%), with an average lesion size of 239 (±218) mm^2^.

**Figure 2:**
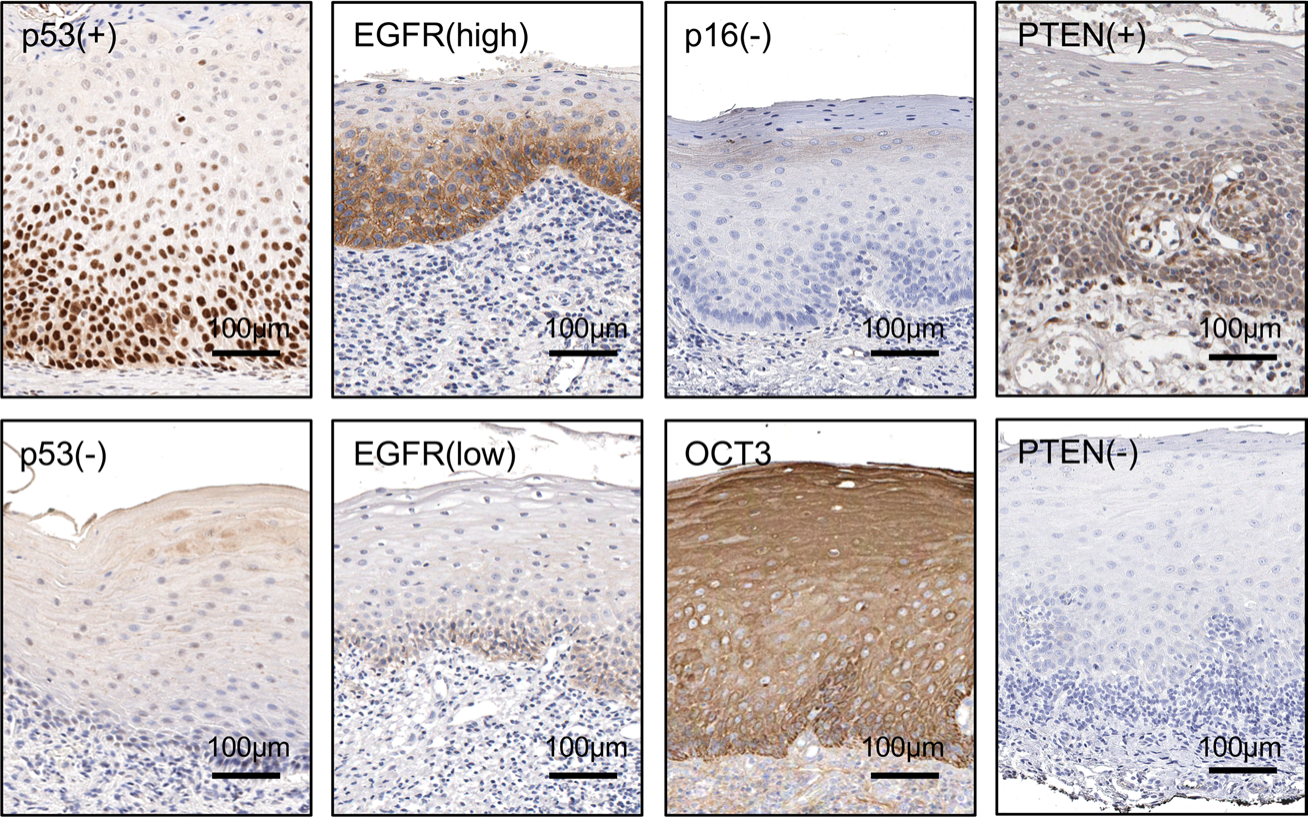
Biomarker analysis. Examples of lesions positive and negative for p53 are shown in the 2 boxes of the first column. In the positive lesions, the expression is limited to the nuclei of the dysplastic proliferating cells. Examples of high and low EGFR expression levels are depicted (second column). All lesions tested negative for p16 and expressed OCT3 throughout the epithelial layers (third column). Examples of positive and negative PTEN lesions are depicted (fourth column). Please see the corresponding data for each individual participant in Table 2.

Subject and OPL characteristics of individuals providing preintervention and postintervention biospecimens are presented per trial participant in Table 2. IHC analysis of the baseline OPLs revealed that 15 of 23 (65%) were positive for nuclear p53 staining, which is used as a surrogate of p53 mutations (33). Example p53^+^ and p53^–^ cases are shown in Figure 2. The high expression of OCT3, a metformin transporter that is widely expressed in normal, dysplastic, and cancerous squamous epithelium (28), was confirmed in all tissues tested. EGFR expression levels were classified as high (27%), moderate (33%), and low (33%) based on the staining intensity. All tissues tested were negative for p16, the cell cycle protein that is upregulated in HPV^+^ HNSCC, lost in the first steps of malignant progression in HPV^–^ HNSCC (34), and used as a surrogate biomarker for HPV status in oropharynx HNSCC. We also examined the expression levels of PTEN, a driver on PI3K/mTOR activation that is often genetically or epigenetically suppressed in HNSCC (35). Only 1 case showed the absence of PTEN immunoreactivity. Thus, most individuals exhibited typical OPLs that are HPV^–^ and p53^+^, expressed the metformin transporter OCT3, and exhibited variable levels of EGFR, with only 1 case exhibiting the lack of expression of PTEN.

**Figure 3:**
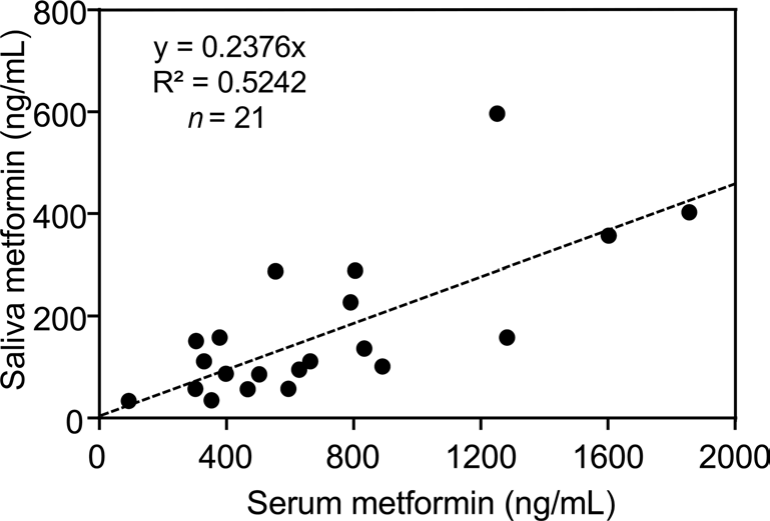
Metformin concentration in blood and saliva and serum biomarkers. Correlation analysis of metformin levels in serum and saliva in individuals treated with metformin.

Baseline and postintervention serum glucose, hemoglobin A1c (HbA1c), and C-peptide concentrations and serum and saliva metformin concentrations are summarized in Table 3, Table 4, and Figure 3. Metformin intervention significantly suppressed serum HbA1c levels (from 5.7 ± 0.5 to 5.5 ± 0.4%, *P* = 0.023) but did not change the glucose and C-peptide levels. The postintervention average serum metformin concentration was 705.0 ± 444.0 ng/mL. Metformin was also detectable in saliva with an average postintervention concentration of 171.0 ± 143.3 ng/mL and was highly correlated with the serum metformin concentration.

Most participants had mild or moderate side effects of metformin (Table 5), all of which were expected. Approximately a third of individuals exhibited gastrointestinal (GI) pain and discomfort, including abdominal pain (2 of 26), bloating (2 of 26), dyspepsia (1 of 26), GI pain (2 of 26), and stomach pain (4 of 26).

**Figure 4:**
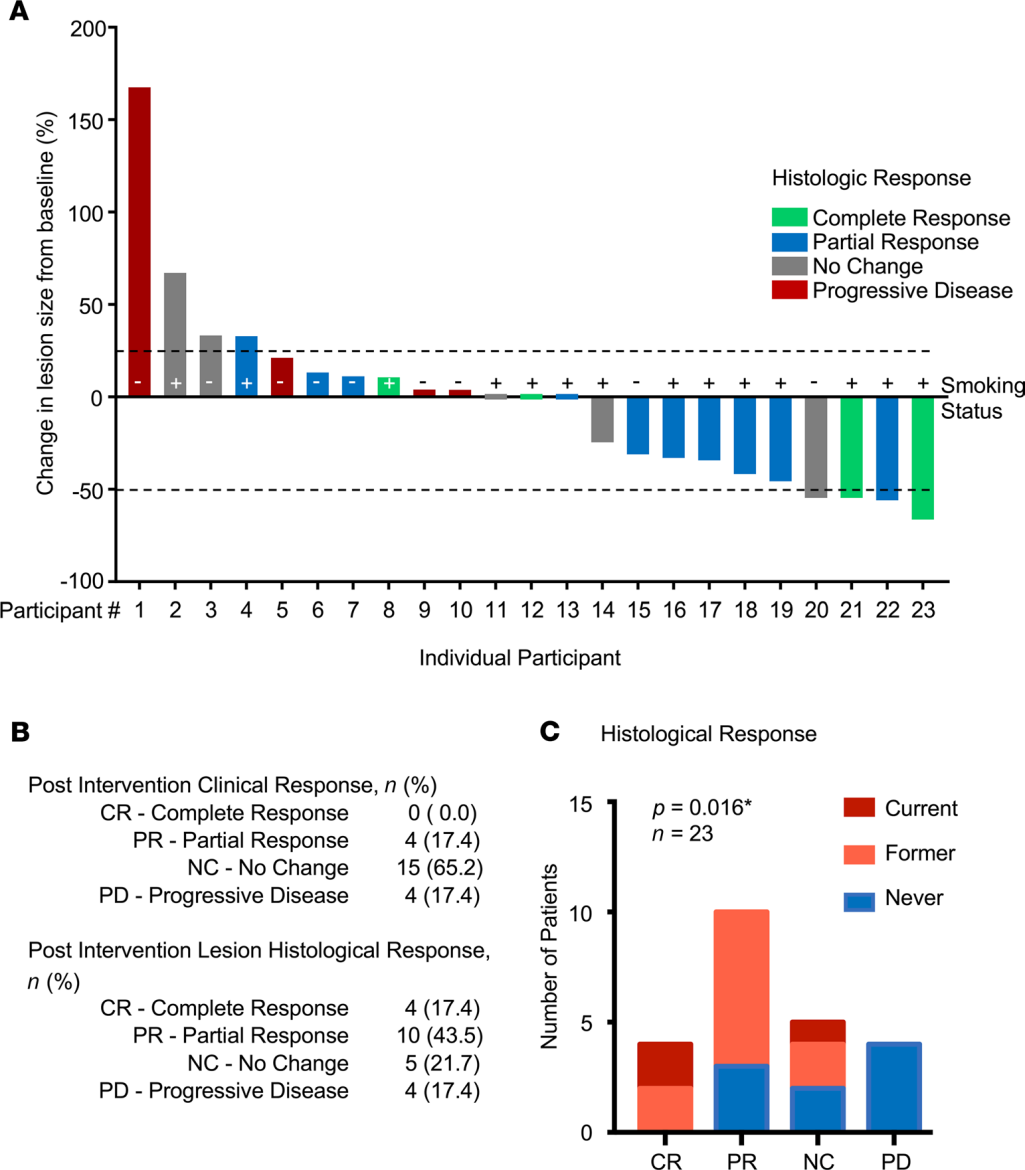
Clinical and histological response to metformin. (**A**) A waterfall plot of the clinical response to treatment is shown, depicting the percentage of change in lesion size. Histological responses are also indicated based on the column color. The individual participant responses as well as the smoking status are depicted following the same numbering as in Figure 1. (**B**) Summary of the clinical and histological responses of all participants evaluated. (**C**) Statistical analysis of the histological response in relationship to the smoking status (*P = 0.016, Fisher’s exact test). CR, complete response; PR, partial response; NC, no change; PD, progressive disease.

The clinical response rate was 17% (1-sided 95% CI: 0.06, 1.00). The waterfall plot depicting changes in the lesion size in individual participants, clinical and histological response, and smoking status is shown in Figure 4A. As detailed in Figure 4B, none of the participants had a complete clinical response; 17% had a partial response and 17% had progressive disease. Of the participants, 17% had a complete histological response and 43% (1-sided 95% CI: 0.26, 1.00) had a partial histological response, for an overall histological response rate of 60%. Of the participants, 17% had progressive disease. Exploratory analysis of the relationship between histological response and smoking status revealed a higher number of responses in current or former smokers versus never smokers (*P* = 0.016; Figure 4C). Notably, all participants who exhibited progressive disease were never smokers.

**Figure 5:**
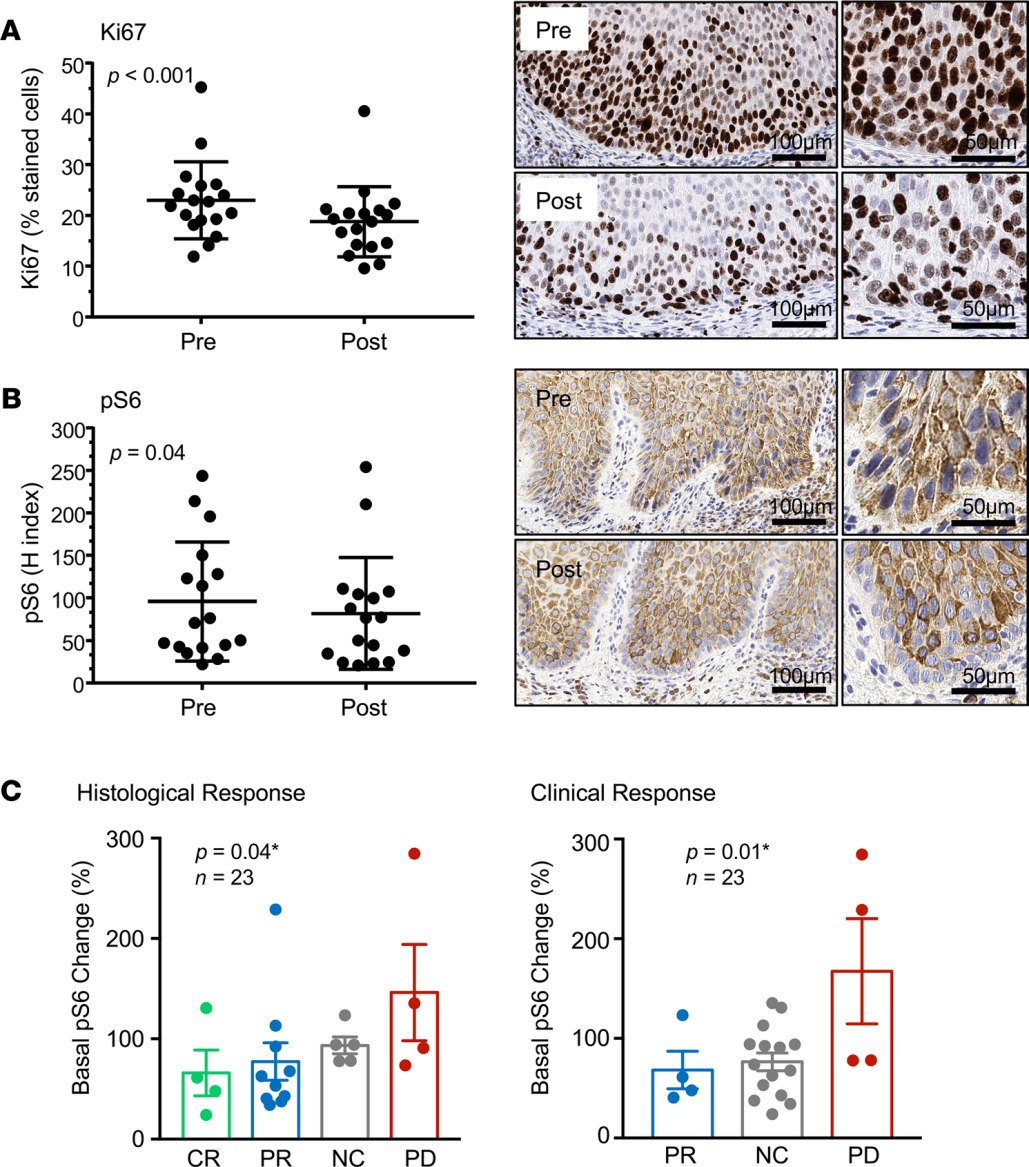
Impact of metformin on proliferation and mTOR pathway signaling in oral premalignant lesions. (**A** and **B**) Quantification of the IHC of the proliferation marker KI67 (**A**) and pS6 (surrogate indicator of the mTOR pathway activity) (**B**) was evaluated and reported as the percentage of positive cells for Ki67 and H score for pS6 before and after metformin treatment. Statistical significance (nonparametric Wilcoxon’s matched-pairs signed-rank test) is indicated, and examples of staining before and after treatment are included in each case, with a higher magnification on the right. Notice the absence of pS6 staining in the basal layer of the oral premalignant lesion after metformin treatment. (**C**) Statistical analysis of the histological (left) and clinical (right) response in relationship to the changes in basal pS6 levels. Statistical significance is indicated (ordinal logistic regression). CR, complete response; PR, partial response; NC, no change; PD, progressive disease.

IHC analysis of cell proliferation (nuclear staining for Ki67) showed that metformin induced a statistically significant decrease in cell proliferation in the squamous epithelium (Figure 5A). Aligned with our prior studies, most OPLs exhibited high levels of mTOR signaling as judged by pS6 staining throughout the lesions, which was reduced significantly by metformin administration (Figure 5B). The expression levels of total S6 protein was not affected, as we previously reported in experimental systems (27–29). Qualitatively, the decrease in pS6 was most notable in the basal epithelial cells (Figure 5B, right panel). Exploratory analysis revealed a significant correlation between the decrease in basal pS6 staining and both histological and clinical response (Figure 5C). In contrast, we did not see any statistically significant changes in the expression levels of the OCT3 metformin transporter or in p53 and EGFR expression (*P* = 0.571, *n* = 20 and *P* = 0.615, *n* = 20, respectively).

As an approach to identify genetic alterations predictive of a favorable response, we performed whole-exome sequencing of the pretreatment OPL biopsies and matching blood DNA. OPLs were small in size and fixed in formalin, making their molecular profiling more challenging. A total of 17 of 22 OPL specimens yielded limited but sufficient DNA to be processed (median 31.2 ng). All 17 samples were analyzed for somatic copy number alterations (CNAs), and 14 of them had more than 70% of their bases covered at 20x and were further analyzed for single-base substitutions.

We identified a total of 1423 mutations, 16 of which were nonsilent, likely pathogenic, and affecting known cancer genes or genes involved in mTOR signaling. These affected 11 of 14 samples and none of them were due to C to A substitution, which could be due to oxidation artifact from FFPE (36). In particular, we identified known or likely pathogenic mutations in *TP53* (*n* = 3), *HRAS* (*n* = 2), *NOTCH1* (*n* = 2), *CDKN2A* (*n* = 1), *PIK3CA* (*n* = 1), or *CASP8* (*n* = 1; Supplemental Table 1; supplemental material available online with this article; https://doi.org/10.1172/jci.insight.147096DS1). This landscape is consistent with mutations identified in HPV^–^ HNSCC (11). None of the mutated genes were significantly associated with treatment response.

The 14 specimens with higher quality data had a median mutational burden of 1.43 single-base substitutions/Mb (a median of 51 exon substitutions). We also determined that a median of 6% of the genome was involved in CNAs, and samples with lower CNA burden were more likely to be from participants responding histologically to treatment (*P* = 0.01, Wilcoxon’s test; Supplemental Figure 2). We identified chromosome arm–level CNAs in 9 of 17 samples, including recurrent copy number gains of 8q (*n* = 3), 8p (*n* = 2), or 9q (*n* = 2) or loss of heterozygosity of 9p (*n* = 5) and 17p (*n* = 4), as well as 4 losses and 3 gains observed in individual samples, some of which have been previously described in OPLs (Supplemental Figure 2, left; ref. 37). None of the arm-level CNAs were significantly associated with response. However, 3 of the 4 individuals with progressive disease exhibited multiple CNAs, including participants 1 and 10 with extensive arm-level and foci-level loss and participant 5 with only arm-level gain. All of these individuals were never smokers. Although numerically less common, the presence of multiple CNAs (loss and/or gain) on several loci appeared to be more frequent in individuals with progressive disease than in responders (3 of 4 vs. 2 of 10), consistent with the CNA burden analysis.

## Discussion

Individuals harboring OPLs are at risk of developing HNSCC. Specifically, OPLs, such as hyperplasia and dysplasia, may undergo variable progression to malignancy over a period of years, thus requiring extended surveillance and possibly therapy (8). OPLs often present as leukoplakia or erythroplakia (white or red patches, respectively), with progression rates to cancer ranging from 11%–36% for leukoplakia to greater than 50% for erythroplakia (38, 39). OPLs are often not resectable due to anatomic location, multifocality, or involvement of broad areas of oral mucosal surfaces. In these cases, long-term surveillance for progression is frequently required for long-term clinical management. Furthermore, even individuals undergoing adequate surgical resection with negative margins have a relatively high rate of progression to HNSCC of 15%–40% (40). This reinforces the concept of epithelial field cancerization and that the presence of OPLs represents a risk for malignant transformation across the oral mucosa due to occult clonal premalignant cells that may demonstrate normal histology (41). Thus, there is an urgency to identify new treatment modalities to intercept the conversion of OPLs into HNSCC. Here, we report the first phase II trial exploring the clinical, histological, and biological activity of metformin in individuals with OPLs. Although the primary endpoint of the clinical response rate (defined as a ≥50% reduction in lesion size) was lower than the proposed threshold for favorable clinical response (≥30%), secondary endpoints, including a 60% histological response rate, tolerability, and evidence of biological activity, encourage further investigation of metformin as a chemopreventive agent for HNSCC.

There is growing enthusiasm for clinical trials using metformin for cancer prevention and treatment. However, there is a limited number of studies using metformin in prospective clinical studies for cancer prevention. The still poorly understood mechanisms of metformin’s purported anticancer activity may also preclude the selection of the patient populations most likely to benefit from metformin treatment. The well-known antihyperglycemic effects of metformin may lower cancer risk by decreasing circulating insulin at the organismal level (42). This may account for the protective effects of metformin in patients with diabetes. However, metformin has also shown chemopreventive efficacy in nondiabetic individuals, where it reduced the prevalence and number of metachronous adenomas or polyps after polypectomy in a phase III randomized, placebo-controlled clinical trial (43). Specifically for HNSCC, metformin displays chemopreventive and antitumor effects in our HNSCC preclinical models in which animals are not obese or insulin-resistant (27–29). Indeed, we have provided evidence that metformin acts on HNSCC-initiating cells directly, because its beneficial effects are dependent on the expression of the metformin transporter OCT3 in HNSCC cells (28) and can be abolished by reverting the impact of metformin on HNSCC mitochondrial complex I (29). The latter approach revealed that in HNSCC, metformin decreases mTOR and AKT activity, activates AMPK, and reduces the expression of cancer stemness gene-expression programs, thereby reducing the proliferative capacity of the precancer cells and enhancing their commitment to terminal differentiation (29). Aligned with these experimental observations, we found a high level of OCT3 expression in OPLs in our current study, and we observed a histological response in 60% of individuals with OPLs after 3 months of treatment with metformin, concomitant with reduced cell proliferation. This included 17% complete pathological responses, without affecting circulating glucose or C-peptide levels and independently of the participants’ BMI. Although the elucidation of the underlying mechanisms may require further investigation, our current findings suggest that metformin may have acted on OPL squamous cells directly, by inhibiting mTOR signaling, reducing cell proliferation, and enhancing cell differentiation toward a more benign or normal histology. In this regard, it is conceivable that longer treatment with metformin would be needed to increase clinical response rates, because this process requires the progressive remodeling of the OPL and its stroma, whereas reduced cell proliferation and histological changes may occur more rapidly.

An unexpected preliminary observation from our study in unselected participants with OPLs is that metformin was more active in current and former smokers, with more histological responses in this particular subgroup. We also observed a numerically lower response rate in never smokers with high levels of CNAs, although these results are to be considered preliminary and hypothesis generating. The effects were independent of the specific gene mutations, albeit only a small number of OPLs yielded high-quality genomic information. Although the mechanistic rationale for these findings is at the present unknown, it is notable that the preclinical activity of metformin in OPL was first revealed in the 4NQO carcinogen-induced, oral-specific carcinogenesis model (27), which we have recently shown to exhibit a mutanome with 94% similarity to the human tobacco-induced carcinogen signature (29). This may have provided a very useful and clinically relevant experimental bias. Specifically, these observations together with the results of our current clinical trial raise the possibility that metformin may have been beneficial primarily in current and former smokers, which is precisely the patient population at the highest risk of HNSCC development (44). This may provide a testable hypothesis by enriching for this high-risk group of participants in future clinical trials. Similarly, we also observed a numerically higher rate of high levels of CNAs in never smokers who progressed on metformin. Although these results are to be considered preliminary and hypothesis generating, CNAs have been associated with immunologically “cold” oral cancers, which could mediate metformin resistance.

A number of limitations to this study must be acknowledged. Notably, there was no randomized placebo control arm to control for the spontaneous rate of OPL clinical and histological regression. The study was powered for a clinical response rate of 30%, as seen in more extended previous OPL clinical trials; however, OPLs typically wax and wane in size, and thus a longer treatment may be necessary to definitively address clinical responses (45, 46). Indeed, the correlation between clinical lesion regression and the lack of cancer development is not well established, because in the setting of oral premalignancy, some cancers can develop outside the target lesion (38). Nevertheless, the goal of this trial was to identify a signal of efficacy, which was realized via the greater-than-expected histological regression rate and the intriguing signal that metformin may be more effective in the setting of tobacco-related field injury (45, 46). The study’s additional strengths include its prospective nature with careful clinical follow-up and sequential biopsies, as well as the thorough tissue analysis of the impact of metformin treatment in previously untreated OPLs. Further study is needed to investigate the efficacy of metformin as a single agent or in rational combinations in a larger, randomized, placebo-controlled trial of longer duration.

In conclusion, metformin is a well-characterized and widely used FDA-approved drug with a known safety profile. Premalignant lesions characterized by upregulated mTOR signaling, such as OPLs, may be uniquely vulnerable to metformin treatment. After only 3 months of treatment, 60% of participants treated with metformin had a partial or complete histological response. OPL tissues showed a reduction in Ki67 expression and phosphorylation of S6, with a marked correlation between the decrease in mTOR signaling in the basal layer of OPLs and both the clinical and histological responses. Overall, the results demonstrate encouraging results and support further study on the potential chemopreventive activity of metformin for HNSCC prevention in selected individuals with OPLs.

## Methods

### Study design.

The study was a phase IIa single-arm, open-label trial of individuals with oral leukoplakia or erythroplakia to explore the potential of metformin for oral cancer prevention (NCT02581137). The primary endpoint was to determine whether 12–14 weeks of metformin intervention is associated with the clinical response of OPLs. The secondary endpoints included histological response to metformin in the target lesion, pretreatment and posttreatment tissue-based biomarkers of molecular targets and dysregulated molecular mechanisms, modulation of circulating metabolic biomarkers, and serum and saliva metformin concentrations.

### Study drug.

The drug product was supplied to the study site by the Division of Cancer Prevention, NCI. The drug product was commercially available metformin hydrochloride extended-release tablets manufactured by Actavis. Each tablet contained 500 mg metformin hydrochloride as the active ingredient. Extended-release metformin was selected for this study to increase compliance and reduce GI side effects.

### Study population.

Study participants were at least 18 years of age, had oral leukoplakia or erythroplakia with mild, moderate, or severe histological dysplasia or hyperplasia not associated with mechanical factors, and had lesions at least 8 × 3 mm before initial biopsy. Other inclusion criteria included Karnofsky performance status greater than or equal to 70%; normal liver, kidney, and bone marrow function; and ability to sign a written informed consent document. Exclusion criteria included the presence of diabetes treated with insulin or oral agents, HbA1c greater than 8%, history of diabetic ketoacidosis, uncontrolled intercurrent illness, oral carcinoma in situ, history of chronic alcohol use or abuse, acute or chronic liver disease, history of renal disease, history of prior HNSCC unless curatively treated 1 year or more prior, and use of chemotherapy and/or radiation for any malignancy (excluding nonmelanoma skin cancer and cancers confined to organs with removal as only treatment) in the past 2 years.

### Study procedures.

During the initial visit, participants underwent a brief physical exam and performance status evaluation. They were also evaluated for concomitant medications, medical history, baseline symptoms and signs, and tobacco and alcohol use. Participants underwent an oral exam for lesion measurement and photography. Bidimensional measurements for all lesions were recorded. All lesions that met the size criteria (≥8 × 3 mm) were considered target lesions for clinical response assessment. Lesions that did not meet the size criteria were also measured but recorded as nonmeasurable.

A biopsy (4 mm) was performed on the lesion that met the size criteria or on the largest lesion, if multiple lesions met the size criteria. The lesions were generally sufficiently large such that the biopsy did not have much effect on lesion size. All biopsies were sent for local pathology evaluation, followed by central pathology review. Archival tissue was used for histological eligibility determination by a centralized pathology review and biomarker analysis if the preenrollment biopsy was performed within 6 weeks of initial screening.

Each of the participating centers performed pathology reviews, and centralized consensus pathology review of the target lesion biopsies was performed at UCSD. A predefined process was developed and followed to resolve discrepancies between the local site and central pathology review. In case of disagreement between the local and central pathology review, the following algorithm was used. For minor discrepancy (1 level change), the in-house pathologist’s evaluation was considered final. For major discrepancy (2 levels or more difference), referral was made for a third independent review (in-house at NCI). The consensus evaluation of 2 pathologists was used as the final diagnosis. For disagreement among all 3 pathologists (that at the local site, UCSD, and NCI), the 2 in-house pathologists discussed the case to come to a consensus.

After eligibility was confirmed, participants were instructed to take metformin extended release 500 mg per day for 1 week, followed by dose escalation to 1000 mg per day for 1 week, followed by dose escalation to 2000 mg per day for the remainder of the study period. Metformin dose escalation is a standard practice in patients with diabetes to minimize the GI side effects and thus optimize adherence. An interim clinic safety visit occurred after 6 weeks of treatment. Participants also underwent an oral exam with lesion measurement and photography and returned after 12–14 weeks of agent intervention for postintervention evaluation. During this visit, participants underwent safety and compliance assessments as well as oral exam for lesion measurement, photography, and biopsy of the previously defined target lesion for histopathology and tissue markers. In general, a biopsy was performed on the residual and worst appearing area of each lesion after treatment. If the target lesion was not visible after intervention, a biopsy was obtained at the site of the previous lesion biopsy. Biofluids were collected for research endpoints.

### Criteria for clinical and histological response evaluation.

Clinical response was evaluated by the following criteria (47): complete response (CR), disappearance of all evidence of lesion(s); partial response (PR), greater than or equal to 50% reduction in the sum of the products of diameters of lesion(s) measurable at baseline; nonmeasurable lesion(s) may not increase greater than or equal to 25% in size and no new lesion may appear; no change (NC), no change in the size of the lesion(s) identified at baseline and no new lesions appearing, i.e., anything that is not CR, PR, or progressive disease (PD); and PD, any increase greater than or equal to 25% in the product of the diameters of any lesion(s) measurable at baseline or in the estimated size of lesion(s) nonmeasurable at baseline or the appearance of an unequivocal new lesion.

Histological response was evaluated by the following criteria: CR, complete reversal of dysplasia or hyperplasia to normal epithelium in the target lesion; PR, improvement of the degree of dysplasia or hyperplasia in the target lesion; NC, no change in the degree of dysplasia or hyperplasia in the target lesion, anything that is not CR, PR, or PD; and PD, increase in the severity of grade of histology in the target lesion.

### Biomarker analysis.

All tissues were fixed overnight in Z-fix (zinc-buffered formaldehyde, Anatech Ltd.), transferred to 70% ethanol, and processed for routine paraffin embedding. Sections (5 μm) were obtained that were stained with H&E for imaging or immunoreacted using the ABC method (Vector Laboratories). A detailed description of the methods used can be found in Supplemental Methods. Quantification of slides stained for different biomarkers was performed using Aperio-Leica Scanscope-associated algorithms. For pS6 IHC, H scores were determined as the product of the staining intensity (0, absent; 1, weak staining; 2, moderate staining; and 3, strong staining) multiplied by the percentage of positive cells quantified. The percentage of cells staining positive for pS6 in the basal layers of OPLs and the percentage of cells positive for OCT3 were also determined. Ki67 quantification was performed using Aperio-Leica Scanscope–associated algorithms, and the percentage of positive cells was determined.

### Analysis of serum and saliva metformin concentrations.

Serum and saliva metformin concentrations were measured using high-performance liquid chromatography–tandem mass spectrometry (HPLC-MS; ref. 48). Briefly, an aliquot of serum or saliva was mixed with the internal standard, phenformin. Cold acetonitrile was added for protein precipitation. The supernatant was injected onto the HPLC-MS system. The mass spectrometric analysis was performed using atmospheric pressure chemical ionization operated in the positive ion mode. The analytes were detected by multiple reaction monitoring. The assay was linear over the concentration range of 2–2000 ng/mL.

### DNA extraction, quality control, sequencing, and copy number analysis.

The DNA was extracted from FFPE tissue using the QIAamp DNA FFPE Tissue Kit (QIAGEN). The extracted DNA was quantified by fluorometry (HS dsDNA Kit Qbit, Thermo Fisher Scientific). A detailed description of the methods used for sequencing and copy number analysis can be found in Supplemental Methods. Individual sequence information was deposited in the National Center for Biotechnology Information’s database of Genotypes and Phenotypes, study phs002437.v1.p1: https://www.ncbi.nlm.nih.gov/projects/gap/cgi-bin/study.cgi?study_id=phs002437.v1.p1

### Statistics.

Participants’ characteristics were summarized by mean ± SD for continuous variables and frequency (%) for categorical variables. The primary objective of this study was to determine whether 12 weeks of treatment with therapeutic doses of metformin was associated with clinical response of the target OPL. Clinical response was evaluated after the 12-week treatment period and categorized into CR, PR, NC, or PD. A participant with CR or PR was considered a respondent. A 1-sided, 1-sample binomial exact test at a significance level of 5% was performed to see if the overall response rate was greater than 30% (i.e., ≤30% considered as poor treatment). A sample size of 20 achieved 87% power to detect a response rate 0.30 higher (i.e., a response rate of 60%) than a poor treatment. With an anticipated attrition rate of 20%, 26 participants were accrued to have at least 20 evaluable participants.

The secondary endpoints included histological response; pretreatment to posttreatment changes in Ki67, pS6, glucose, and C-peptide; and the effect of OCT3 expression level and genomic alterations on biomarker modulation by metformin treatment and clinical or histological response to metformin. Similar to the primary endpoint, the histological response rate was calculated, and a 1-sided 95% CI based on the exact (Clopper-Pearson) method was derived. Nonparametric methods, e.g., signed-rank test, were performed to evaluate each of the biomarker changes. In addition, logistic regression with the clinical/histological response as the outcome variable was performed to explore if smoking status, changes in pS6 expression, changes in OCT3 expression level, or any genomic alteration was associated with the clinical/histological response to metformin. Statistical analyses were performed using SAS 9.4. The nonparametric Wilcoxon’s matched-pairs signed-rank test was used to compare pretreatment and posttreatment values of IHC stainings using GraphPad Prism version 7.00 for Windows.

### Study approval.

The study was conducted at the UCSD Moores Cancer Center, UMN, and BCCA through the University of Arizona Chemoprevention Consortium and funded by the Division of Cancer Prevention, NCI. The study was approved by the NCI Central Institutional Review Board and IRBs at each institution. Written informed consent was received from participants prior to inclusion in the study.

## Author contributions

JSG and ES initiated, designed, and directed the research studies; analyzed the data; and wrote the manuscript. AAM performed the central pathology review and IHC studies, and SMH reviewed pathology and contributed with methods development. XW and ZW collected samples, performed biochemical analysis, and contributed to statistical analysis. DN, OH, and LBA conducted the sequencing and genetic analysis of OPLs. BRW, FGO, DL, LDR, MR, and CC identified the participants; conducted the biopsies, treatment, and subject follow-up; and acquired the data. VDB and LB coordinated the trial at all sites. CHH conducted the statistical design and analysis and wrote the corresponding part of the manuscript. EEWC and SML contributed to the trial design and implementation. SML was the trial principal investigator. JEB and HHSC contributed to the trial design and implementation. HHSC had overall responsibility of the trial conduct and coordination and measurement of metformin concentrations.

## Supplementary Material

Supplemental data

## Figures and Tables

**Table 5:**
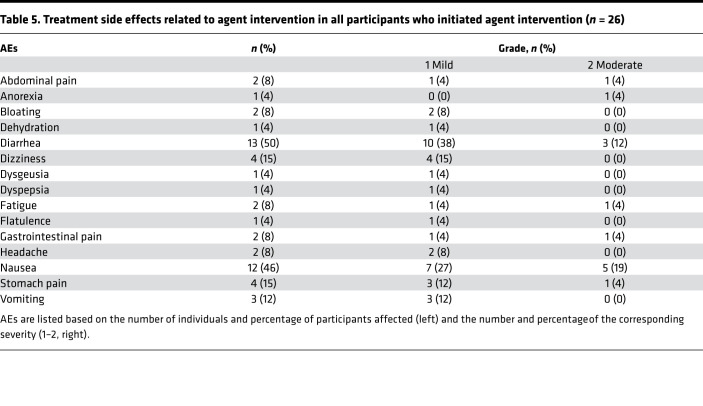
Treatment side effects related to agent intervention in all participants who initiated agent intervention (*n* = 26)

**Table 4:**
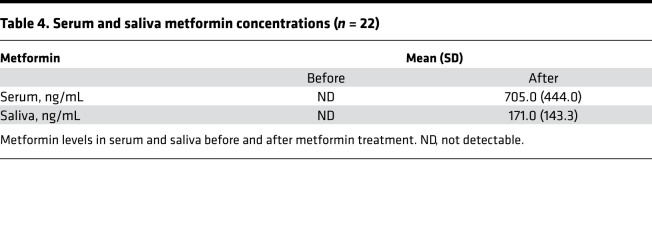
Serum and saliva metformin concentrations (*n* = 22)

**Table 1:**
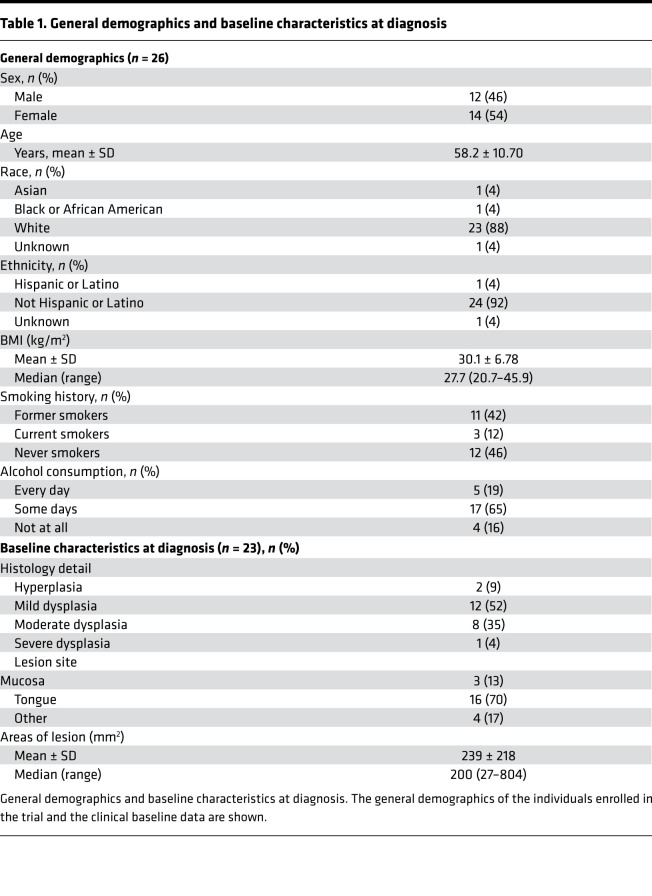
General demographics and baseline characteristics at diagnosis

**Table 2:**
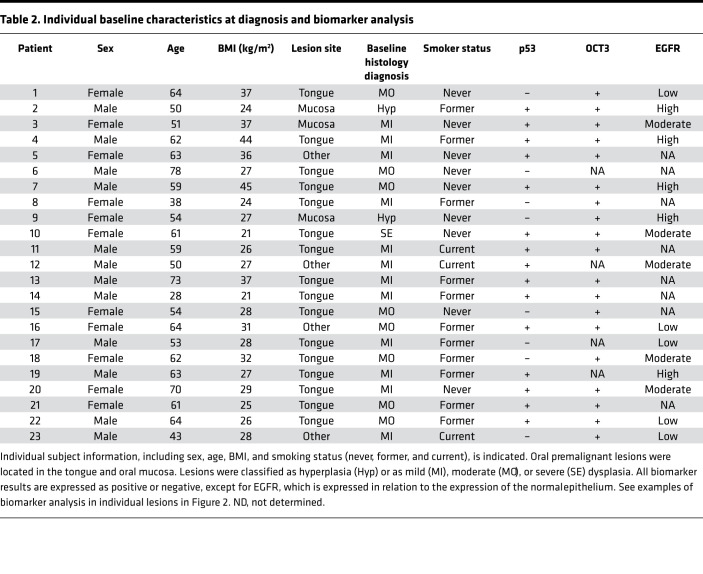
Individual baseline characteristics at diagnosis and biomarker analysis

**Table 3:**
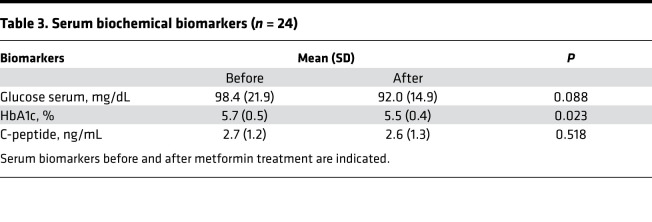
Serum biochemical biomarkers (*n* = 24)

## Version 1 07/13/2021

In-Press Preview

## Version 2 09/08/2021

Electronic publication

## References

[1] Siegel RL (2020). Cancer statistics, 2020. CA Cancer J Clin.

[2] Pulte D, Brenner H (2010). Changes in survival in head and neck cancers in the late 20th and early 21st century: a period analysis. Oncologist.

[3] Slaughter DP (1953). Field cancerization in oral stratified squamous epithelium; clinical implications of multicentric origin. Cancer.

[4] Hong WK (1986). 13-cis-retinoic acid in the treatment of oral leukoplakia. N Engl J Med.

[5] Papadimitrakopoulou VA (2009). Randomized trial of 13-cis retinoic acid compared with retinyl palmitate with or without beta-carotene in oral premalignancy. J Clin Oncol.

[6] William WN (2016). , et al. Erlotinib and the risk of oral cancer: the erlotinib prevention of oral cancer (EPOC) randomized clinical trial. JAMA Oncol.

[7] Bauman JE, Grandis J (2016). Oral cancer chemoprevention-the end of EPOC, the beginning of an epoch of molecular selection. JAMA Oncol.

[8] Warnakulasuriya S (2008). Oral epithelial dysplasia classification systems: predictive value, utility, weaknesses and scope for improvement. J Oral Pathol Med.

[9] Iglesias-Bartolome R (2013). Exploiting the head and neck cancer oncogenome: widespread PI3K-mTOR pathway alterations and novel molecular targets. Cancer Discov.

[10] Lui VWY (2013). Frequent mutation of the PI3K pathway in head and neck cancer defines predictive biomarkers. Cancer Discov.

[11] Cancer Genome Atlas Network (2015). Comprehensive genomic characterization of head and neck squamous cell carcinomas. Nature.

[12] Molinolo AA (2007). Dissecting the Akt/mammalian target of rapamycin signaling network: emerging results from the head and neck cancer tissue array initiative. Clin Cancer Res.

[13] Molinolo AA (2012). mTOR as a molecular target in HPV-associated oral and cervical squamous carcinomas. Clin Cancer Res.

[14] Raimondi AR (2009). Rapamycin prevents early onset of tumorigenesis in an oral-specific K-ras and p53 two-hit carcinogenesis model. Cancer Res.

[15] Czerninski R (2009). Targeting mammalian target of rapamycin by rapamycin prevents tumor progression in an oral-specific chemical carcinogenesis model. Cancer Prev Res (Phila).

[16] Patel V (2011). Decreased lymphangiogenesis and lymph node metastasis by mTOR inhibition in head and neck cancer. Cancer Res.

[17] Saito K (2012). Longitudinal imaging studies of tumor microenvironment in mice treated with the mTOR inhibitor rapamycin. PLoS One.

[18] Squarize CH (2008). Chemoprevention and treatment of experimental Cowden’s disease by mTOR inhibition with rapamycin. Cancer Res.

[19] Sun Z-J (2012). Chemopreventive and chemotherapeutic actions of mTOR inhibitor in genetically defined head and neck squamous cell carcinoma mouse model. Clin Cancer Res.

[20] Day TA (2019). Inhibition of mTOR signaling and clinical activity of Rapamycin in head and neck cancer in a window of opportunity trial. Clin Cancer Res.

[21] Cohen EE (2012). Phase I studies of sirolimus alone or in combination with pharmacokinetic modulators in advanced cancer patients. Clin Cancer Res.

[22] Viollet B (2012). Cellular and molecular mechanisms of metformin: an overview. Clin Sci (Lond).

[23] Pollak M (2010). Metformin and other biguanides in oncology: advancing the research agenda. Cancer Prev Res (Phila).

[24] Quinn BJ (2013). Repositioning metformin for cancer prevention and treatment. Trends Endocrinol Metab.

[25] Pollak MN (2012). Investigating metformin for cancer prevention and treatment: the end of the beginning. Cancer Discov.

[26] Pierotti MA (2013). Targeting metabolism for cancer treatment and prevention: metformin, an old drug with multi-faceted effects. Oncogene.

[27] Vitale-Cross L (2012). Metformin prevents the development of oral squamous cell carcinomas from carcinogen-induced premalignant lesions. Cancer Prev Res (Phila).

[28] Madera D (2015). Prevention of tumor growth driven by PIK3CA and HPV oncogenes by targeting mTOR signaling with metformin in oral squamous carcinomas expressing OCT3. Cancer Prev Res (Phila).

[29] Wang Z (2019). Syngeneic animal models of tobacco-associated oral cancer reveal the activity of in situ anti-CTLA-4. Nat Commun.

[30] Tseng CH (2016). Metformin may reduce oral cancer risk in patients with type 2 diabetes. Oncotarget.

[31] Yen YC (2015). Effect of metformin on the incidence of head and neck cancer in diabetics. Head Neck.

[32] Sandulache VC (2014). Association between metformin use and improved survival in patients with laryngeal squamous cell carcinoma. Head Neck.

[33] Taylor D (1999). Immunohistochemical detection of p53 protein accumulation in head and neck cancer: correlation with p53 gene alterations. Hum Pathol.

[34] Adelstein DJ (2009). Head and neck squamous cell cancer and the human papillomavirus: summary of a National Cancer Institute State of the Science Meeting, November 9–10, 2008, Washington, DC. Head Neck.

[35] Izumi H (2020). Pathway-specific genome editing of PI3K/mTOR tumor suppressor genes reveals that PTEN loss contributes to cetuximab resistance in head and neck cancer. Mol Cancer Ther.

[36] Costello M (2013). Discovery and characterization of artifactual mutations in deep coverage targeted capture sequencing data due to oxidative DNA damage during sample preparation. Nucleic Acids Res.

[37] Wood HM (2017). The genomic road to invasion-examining the similarities and differences in the genomes of associated oral pre-cancer and cancer samples. Genome Med.

[38] Lee JJ (2000). Predicting cancer development in oral leukoplakia: ten years of translational research. Clin Cancer Res.

[39] Reichart PA, Philipsen HP (2005). Oral erythroplakia--a review. Oral Oncol.

[40] Arnaoutakis D (2013). Recurrence patterns and management of oral cavity premalignant lesions. Oral Oncol.

[41] Braakhuis BJ (2005). Expanding fields of genetically altered cells in head and neck squamous carcinogenesis. Semin Cancer Biol.

[42] Pollak M (2012). The insulin and insulin-like growth factor receptor family in neoplasia: an update. Nat Rev Cancer.

[43] Higurashi T (2016). Metformin for chemoprevention of metachronous colorectal adenoma or polyps in post-polypectomy patients without diabetes: a multicentre double-blind, placebo-controlled, randomised phase 3 trial. Lancet Oncol.

[44] Hashibe M (2009). Interaction between tobacco and alcohol use and the risk of head and neck cancer: pooled analysis in the International Head and Neck Cancer Epidemiology Consortium. Cancer Epidemiol Biomarkers Prev.

[45] Armstrong WB (2013). Bowman birk inhibitor concentrate and oral leukoplakia: a randomized phase IIb trial. Cancer Prev Res (Phila).

[46] Papadimitrakopoulou VA (2008). Pilot randomized phase II study of celecoxib in oral premalignant lesions. Clin Cancer Res.

[47] Lippman SM (1993). Comparison of low-dose isotretinoin with beta carotene to prevent oral carcinogenesis. N Engl J Med.

[48] Nguyen MM (2018). Bioactivity and prostate tissue distribution of metformin in a preprostatectomy prostate cancer cohort. Eur J Cancer Prev.

