# Supplementary material for: Inhibition of mTOR signaling and clinical activity of metformin in oral premalignant lesions
Source: JCI Insight. 2021 Sep 8;6(17):e147096. doi: 10.1172/jci.insight.147096 (PMC8492350; doi:10.1172/jci.insight.147096)
Supplement: Supplemental data [file jciinsight-6-147096-s149.pdf]

Supplemental Table 1. Mutated genes and histological response

Subject number	Gene	Amino-acid change	Predicted effect	Histologic Response
18	*CASP8*	p.Trp479*	Putative lof	PR
10	*CDKN2A*	p.Ala138Val	Likely lof	PD
5	*HRAS*	p.Gly13Asp	Likely gof	PD
18	*HRAS*	p.Gly12Asp	Likely gof	PR
9	*NOTCH1*	p.Ala465Thr	Likely lof	PD
6	*NOTCH1*	p.Ala465Thr	Likely lof	PR
6	*PIK3CA*	p.Asn1068fs	Likely gof	PR
20	*TP53*	p.Arg273Pro	Known lof	NC
15	*TP53*	p.Ile195fs	Known lof	PR
17	*TP53*	p.Arg196*	Known lof	PR

Supplemental Figure 2.
Copy number alteration and histological response

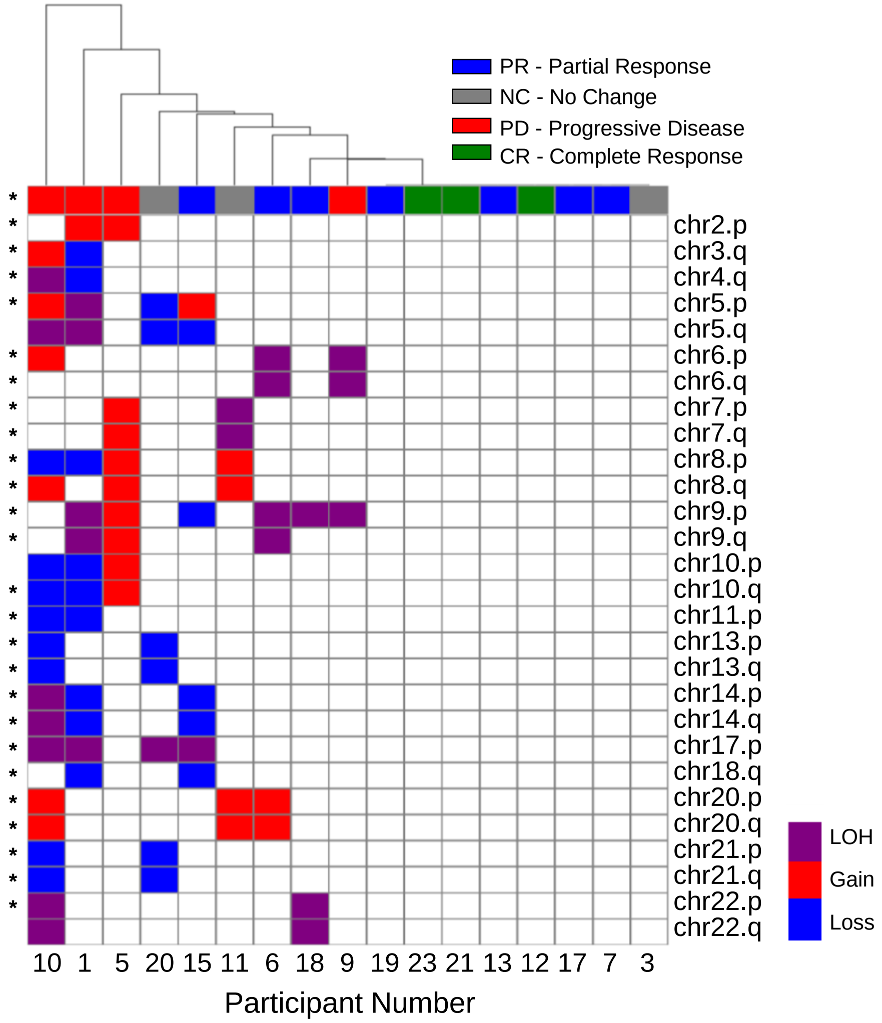

Supplementary Information

Biomarker analysis
All tissues were fixed overnight in Z-fix (zinc buffered formaldehyde, Anatech Ltd.), transferred to 70% ethanol, and processed for routine paraffin embedding. Five µm sections were obtained that were stained with H&E for imaging or immunoreacted using the ABC method (Vector). All slides were scanned with CS Scanscope (Leica/Aperio, Vista, CA). For imaging and diagnostic purposes, the slides were dewaxed in SafeClear II (Fisher Scientific), and hydrated through graded alcohols to water. For immunohistochemistry (IHC) the tissue slides were dewaxed in SafeClear II (Fisher Scientific), hydrated through graded alcohols, and microwaved in pH6.0, 10mM citrate acid for antigen unmasking. The endogenous peroxidase activity was quenched by incubation in with 5% H_2O_2 diluted in 70% alcohol for 30 min. The tissues then washed several times with distilled water, washed 3 times with PBS, and blocked for 30 min with 2.5 % bovine serum albumin in PBS. All primary antibodies were incubated overnight at 4°C. When required, blocking solution was used as diluent. The slides were washed in PBS three times, incubated with a biotin-conjugated secondary antibody in blocking solution at room temperature for 30 minutes, followed by the avidin-biotin complex (Vector Stain Elite Standard, ABC kit, Vector Laboratories) for 30 minutes at room temperature. The slides were washed and developed in 3,3′-diaminobenzidine (Sigma FASTDAB tablet, Sigma Chemical) under microscopic observation. The reaction was stopped in tap water and the tissues were counterstained with hematoxylin, dehydrated, and mounted. Primary antibodies used were: EGFR, EGFR pharmDx, Dako K1492, mouse monoclonal (prediluted); Ki-67, Dako M7240, mouse monoclonal anti-human (1:100); pS6, Cell Signaling 2211S, rabbit polyclonal (1:200); p53, Biocare Medical PM042AA mouse monoclonal (prediluted); p16, p16 CINtec Histology, mouse monoclonal (prediluted); and PTEN, Biocare Medical PM042AA, mouse monoclonal (prediluted). Secondary antibodies used were: ABC Kit Elite, Universal (Vector); Anti-mouse IgG (H+L), (Vector) (1:400); and Anti-rabbit IgG (H+L), (Vector) (1:400). Quantification of slides stained for different biomarkers was performed using Aperio-Leica Scanscope-associated algorithms. For pS6 IHC H-scores were determined as the product of the staining intensity (0, absent; 1, weak staining; 2, moderate staining; and 3, strong staining) multiplied by the percentage of positive cells quantified. The % of pS6 positive stained cells in the basal layer of OPL was also determined, as well as the % of cells positive for OCT3. Ki67 quantification was performed using Aperio-Leica Scanscope-associated algorithms, and the % of positive cells determined.

DNA extraction and quality control (QC).
The DNA was extracted from FFPE tissue using the QIAamp DNA FFPE tissue kit (Qiagen). The extracted DNA was quantified by fluorometry (HS dsDNA kit Qbit – Thermofisher).

Whole exome capture and sequencing
The DNA was sheared down to 200 bp using adaptive focused acoustic on the Covaris E220 (Covaris Inc) following manufacturer recommendations using 50µL of Low TE buffer in microTUBE-130 tubes. Libraries were prepared with the SureSelect XT HS protocol (Agilent Technologies) extending the adapter ligation time to 45 min. After ligation, excess adapters were removed using a 0.8x SPRI bead clean up with Agencourt AMPure XP beads (Beckman Coulter), then eluted into 21 µL of nuclease-free water. Samples were paired and combined (12µL total) to yield a capture "pond" of at least 350 ng, and supplemented with 5µL of SureSelect XTHS and XT Low Input Blocker Mix. The baits for target enrichment consisted of Human All Exon V7 panel (S31285117). The hybridization and capture was performed using Agilent SureSelect XT HS Target Enrichment Kit following manufacturer's recommendations. Post-capture amplification was performed on the beads in a 25µL reaction: 12.5µL of nuclease-free water, 10µL 5x Herculase II Reaction Buffer, 1µL Herculase II Fusion DNA Polymerase, 0.5µL 100mM dNTP Mix and 1µL

SureSelect Post-Capture Primer Mix. The reaction was denatured for 30 sec at 98°C, then amplified for 12 cycles of 98°C for 30 sec, 60°C for 30 sec and 72°C for 1 min, followed by an extension at 72°C for 5 minutes and a final hold at 4°C. Libraries were purified with a 1x AMPure XP bead clean up and eluted into 20µL nuclease free water in preparation for sequencing. The resulting libraries were analyzed using the Agilent 4200 Tapestation (D1000 ScreenTape) and quantified by fluorescence (Qbit – ThermoFisher). The libraries with distinct indexes were pooled in equimolar amounts. All libraries were then sequenced using the HiSeq 4000 sequencer (Illumina) for 100 cycles in paired-end mode. The libraries were later demultiplexed using bcl2fastq software. Individual sequence information was deposited in dgGAP, study phs2437.v1.

Sequencing reads processing and coverage quality control
Sequencing data was analyzed using bcbio-nextgen (v1.1.6) as a workflow manager (bcbio-nextgen, https://github.com/chapmanb/bcbio-nextgen). Adapter sequences were trimmed using Atropos (v1.1.22) (1), the trimmed reads were subsequently aligned with bwa-mem (v0.7.17) to reference genome hg19, then PCR duplicates were removed using biobambam2 (v2.0.87). Additional BAM file manipulation and collection of QC metrics was performed with picard (v2.20.4) and samtools (v1.9).

Somatic variant calling
Somatic variants were called on 14/17 sequenced lesions which had sufficient coverage, (>70% of targeted bases covered at least 20X). Somatic single nucleotide variants (SNVs) and short insertions and deletions (indels) were determined using Mutect2(v2.2), VarDictJava (v1.6.0), VarScan (v2.4.3) and Freebayes (v1.1.0.46) using matched subject blood samples to remove germline variants (2, 3). For SNVs, only variants called by all 4 algorithms were considered, while for indels 3 of 4 were required including VarDict. Variants were required to fall within the boundaries of targeted regions. Functional effects were predicted using SnpEff (v4.3.1) (4). Candidate somatic SNVs were further filtered for high-quality variants: Mutect2's somatic probability score (TLOD) greater than 12, Fisher strand bias phred-scaled p-value greater than 10 and variant allelic fraction (VAF) greater than 0.1. Candidate somatic indels were filtered for VarDict's SSF score lower than 0.05, microsatellite length lower than 5 and VAF greater than 0.05. Genomic burden was estimated for each sample by dividing the total somatic mutations by the total number of targeted bases. Likely pathogenic mutations in candidate genes were identified as nonsense, splice-site, frameshift or missense mutations predicted to be deleterious (CADD score > 20) or annotated as gain or loss of function in OncoKB database (5, 6).

Copy number analysis
Copy number alterations (CNAs) were called were called using allele-specific copy number calling algorithm ASCAT (7) on paired OPL and normal blood bam files. ASCAT was run with default parameters with the exception of a segmentation penalty of 100 and a gamma of 1. Chromosomal arm gains, losses and copy neutral loss of heterozygosity (LOH) were called when more than half their total length was involved in a gained, loss or LOH segment, respectively.

Supplemental Table 1. Mutated genes and histological responses in oral premalignant lesions. Typical mutations observed in head and neck cancer patients that were identified in each indicated oral premalignant lesion are shown, including the gene name, amino acid change, predicted loss of function (lof) or gain of function (gof) of each mutation, and the histological response of each corresponding subject.

Supplemental Figure 1. Impact of metformin on OCT3 expression in oral premalignant lesions. Quantification of the immunohistochemistry evaluations of the % of stained cells with

OCT3 in the epithelial layers. Examples of staining pre- and post- treatment are included. No significant (ns) changes in the expression of OCT3 were observed.

Supplemental Figure 2. Genomic alterations in oral premalignant lesions. Copy number alteration and histological response. Chromosomal arm gains (red) or losses (blue) or copy neutral loss of heterozygosity (purple) found in at least 2 individuals is indicated, as well as the histological response after metformin treatment.

Bibliography

1. Didion JP, Martin M, and Collins FS. Atropos: specific, sensitive, and speedy trimming of sequencing reads. *PeerJ.* 2017;5(e3720.
2. Cibulskis K, Lawrence MS, Carter SL, Sivachenko A, Jaffe D, Sougnez C, Gabriel S, Meyerson M, Lander ES, and Getz G. Sensitive detection of somatic point mutations in impure and heterogeneous cancer samples. *Nat Biotechnol.* 2013;31(3):213-9.
3. Lai Z, Markovets A, Ahdesmaki M, Chapman B, Hofmann O, McEwen R, Johnson J, Dougherty B, Barrett JC, and Dry JR. VarDict: a novel and versatile variant caller for next-generation sequencing in cancer research. *Nucleic acids research.* 2016;44(11):e108.
4. Cingolani P, Platts A, Wang le L, Coon M, Nguyen T, Wang L, Land SJ, Lu X, and Ruden DM. A program for annotating and predicting the effects of single nucleotide polymorphisms, SnpEff: SNPs in the genome of Drosophila melanogaster strain w1118; iso-2; iso-3. *Fly.* 2012;6(2):80-92.
5. Kircher M, Witten DM, Jain P, O'Roak BJ, Cooper GM, and Shendure J. A general framework for estimating the relative pathogenicity of human genetic variants. *Nature genetics.* 2014;46(3):310-5.
6. Chakravarty D, Gao J, Phillips SM, Kundra R, Zhang H, Wang J, Rudolph JE, Yaeger R, Soumerai T, Nissan MH, et al. OncoKB: A Precision Oncology Knowledge Base. *JCO precision oncology.* 2017;
7. Raine KM, Van Loo P, Wedge DC, Jones D, Menzies A, Butler AP, Teague JW, Tarpey P, Nik-Zainal S, and Campbell PJ. ascatNgs: Identifying Somatically Acquired Copy-Number Alterations from Whole-Genome Sequencing Data. *Current protocols in bioinformatics.* 2016;56(15 9 1- 9 7.
